# Pediatric Primary Alveolar Soft Part Sarcoma of the Bladder

**DOI:** 10.1155/2018/1284756

**Published:** 2018-11-26

**Authors:** Kazuya Tanabe, Shigeru Nakamura, Taiju Hyuga, Shina Kawai, Masahiro Yamazaki, Yohei Kawashima, Rieko Furukawa, Toshiro Niki, Shigeru Ono, Hideo Nakai

**Affiliations:** ^1^Department of Pediatric Urology, Jichi Medical University, Children's Medical Center, Tochigi, Japan; ^2^Department of Urology, Jichi Medical University, Tochigi, Japan; ^3^Department of Urology, Ohta Nishinouchi Hospital, Fukushima, Japan; ^4^Department of Pediatric Medical Imaging, Jichi Medical University, Tochigi, Japan; ^5^Department of Diagnostic Pathology, Jichi Medical University, Tochigi, Japan; ^6^Department of Pediatric Surgery, Jichi Medical University, Children's Medical Center, Tochigi, Japan

## Abstract

A 9-year-old girl was diagnosed with primary alveolar soft part sarcoma of the bladder after imaging examinations and transurethral resection (TUR) of the bladder tumor. As a positive surgical margin of the TUR indicated residual tumor cells, we performed a cystourethrectomy to remove the tumor. A continent urinary reservoir for self-catheterization was constructed using the Mainz pouch technique, and an abdominal (umbilical) continent catheterizable stoma using the appendix was performed. For 2.5 years postoperatively, the patient remained free of local recurrence and distant metastasis. The patient's clinical course has been favorable, with good management of clean intermittent self-catheterization.

## 1. Introduction

Alveolar soft part sarcoma (ASPS) is an exceptionally rare disease among soft tissue sarcoma cases [1]. Only four adult cases of primary ASPS of the bladder have been reported [2–5], and there is no reported pediatric case to the best of our knowledge. We report here our experience with a pediatric case of primary ASPS of the bladder.

## 2. Case Presentation

**Figure 1:**
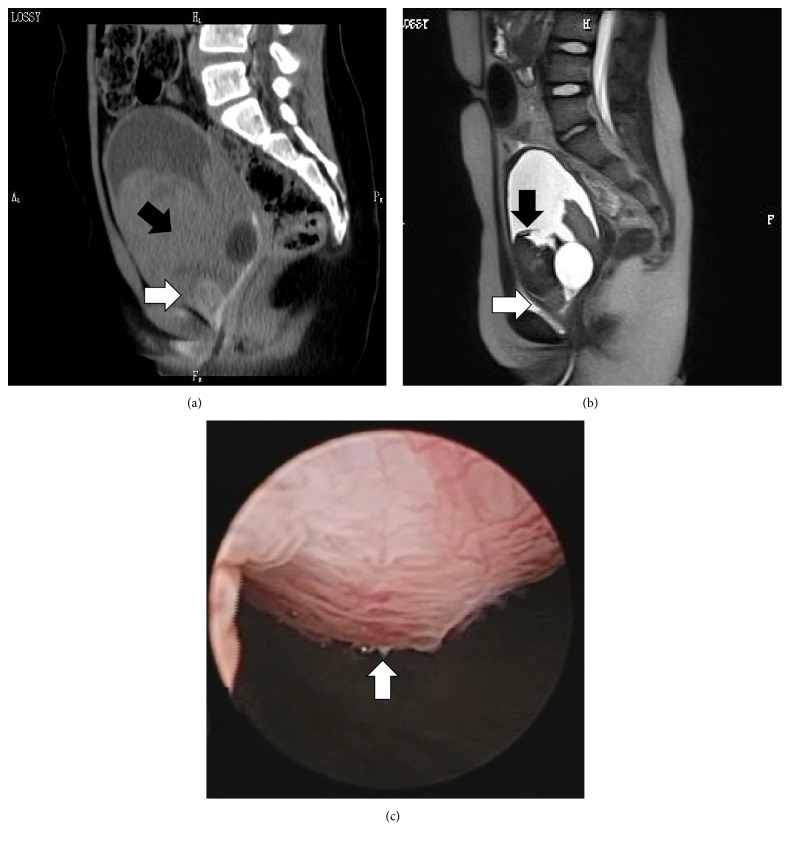
(a) A high-density lesion (sized 10 mm) is observed in the bladder neck on plain computed tomography images (indicated by a white arrow). A hematoma in the bladder is also observed (indicated by a black arrow). (b) A high-intensity lesion (sized 10 mm) is observed in the bladder neck on plain magnetic resonance T2-weighted images (indicated by a white arrow). A hematoma in the bladder is also observed (indicated by a black arrow). (c) A sessile nodular tumor with a smooth surface (sized 10 mm) located in the direction of 10 o'clock in the bladder neck is observed with cystoscopy (indicated by a white arrow).

**Figure 2:**
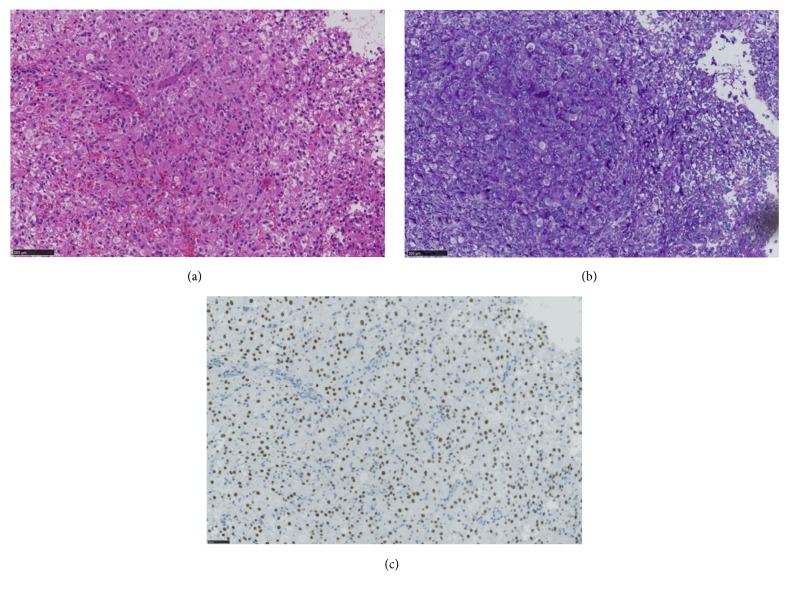
(a) Alveolar-structured intense proliferation of tumor cells with a large acidophilic cytoplasm accompanied by abundant capillary vessels is observed with hematoxylin and eosin (H&E) staining. (b) The tumor cells are positive for periodic acid-Schiff (PAS) staining. (c) The tumor cells with pachychromatic nuclei are observed with TFE3 staining.

A 9-year-old girl visited a local clinic with a major complaint of asymptomatic macroscopic hematuria. Her height was 129 cm and weight was 29 kg. No particular family/medical/birth history was noted. Results of biochemical tests and urine analyses were within the normal range, and urine cytology showed class I. Plain computed tomography (CT) (Figure 1(a)) and plain magnetic resonance imaging (MRI) (Figure 1(b)) showed a 10-mm nodular tumor in the bladder neck, located in the direction of 10 o'clock. The patient was diagnosed with bladder tumor, and transurethral resection of the bladder tumor (TUR-Bt) was performed in the local clinic (Figure 1(c)). Histopathological tests showed alveolar-structured intense proliferation of tumor cells with a large acidophilic cytoplasm accompanied by abundant capillary vessels (Figure 2(a)). Tumor cells were positive for periodic acid-Schiff (PAS) (Figure 2(b)), but negative for PAS after diastase digestion. Immunostaining results were positive for TFE3 (Figure 2(c)), smooth muscle actin, MyoD1, and p53, while they were negative for HMB45, melan A, S100, CD1a, desmin, h-caldesmon, myogenin, myoglobin, EMA, and CAM5.2. Muscular, epithelial, and melanoma markers were negative. According to those findings, the patient was diagnosed with ASPS. As a positive surgical margin of TUR indicated residual tumor cells, the patient was referred to our children's hospital for more detailed investigation and specialized care. Additional examinations performed in our hospital including cystoscopy, contrast-enhanced CT, contrast-enhanced MRI, and positron emission tomography (PET) did not indicate any obvious residual tumor in the bladder. However, after careful consideration regarding a therapeutic strategy, we determined that cystourethrectomy was indicated due to the possibility of residual tumor in the bladder. For urinary diversion, construction of a continent urinary reservoir for self-catheterization (Mainz pouch technique) and the abdominal (umbilical) continent catheterizable stoma using the appendix were selected. No residual tumor was observed macroscopically or histopathologically in the removed bladder. The patient's clinical course after surgery has been favorable. She performs clean intermittent self-catheterization using the abdominal (umbilical) continent catheterizable stoma six times per day, and to date, no urinary incontinence or stricture of the catheterization route has been observed. The patient is under the postoperative follow-up observation with triannual contrast-enhanced CT and annual PET scan. To date, for 2.5 years postoperatively, the patient has been free of any local recurrence or distant metastasis of sarcoma.

## 3. Discussion

ASPS is an exceptionally rare disease accounting for only 0.5-1% of soft tissue sarcoma cases, with the lower extremity being a common site of the primary [6]. The most frequent age of onset is 15-35 years, and it is reported as relatively common in women [6]. Progression of ASPS is generally slower than other types of soft tissue sarcoma and the prognosis has been considered relatively favorable but the cases with distant metastasis are unfavorable. According to a previous report, survival rates of 2, 5, 10, and 20 years are 87%, 62%, 43%, and 18%, respectively [6].

As the primary local lesion is often asymptomatic, ASPS is likely to be detected owing to the subjective symptoms associated with a metastatic lesion, and the prognosis of such cases with distant metastasis is usually poor [6]. Distant metastasis is most likely to occur in the lung (38%), the bone (33%), and the brain (32%), and the mean survival duration after metastasis development is 2 years [6].

The present article is the first report of a pediatric case of primary ASPS of the bladder. After this patient was histopathologically diagnosed with ASPS based on the results from TUR-Bt, cystourethrectomy was performed to completely remove the tumor due to the possibility of residual tumor in the bladder. The progress of the patient has been favorable for 2.5 years postoperatively, without any recurrence or metastasis.

Table 1 summarizes the four adult cases of primary ASPS of the bladder previously reported. As with our case, all adult cases were detected by the development of urinary symptoms. Surgical treatment was selected for three of the cases, but treatment details were unspecified for case 2. Regarding distant metastasis, the lesions for cases 3 and 4 were locally restricted in the bladder and no distant metastasis or recurrence occurred during the follow-up period; details are unknown for cases 1 and 2. Local symptoms of primary ASPS of the bladder, such as urinary symptoms (e.g., macroscopic hematuria), develop at an earlier stage than primary ASPS of other organs. In addition, ASPS has a slowly progressive nature [6]. Therefore, we assumed that early detection and treatment may be possible before development of the distant metastasis. As residual tumor was not histopathologically observed in the removed bladder, cystourethrectomy may have been deemed overtreatment. For ASPS with residual tumor, complete resection of the tumor improves the patient's prognosis [7].

As shown in Table 1, there has been no reported treatment option other than surgery. Considering curability as the first priority, we believe that cystourethrectomy was an appropriate treatment strategy in this case.

The first-line choice for treating primary ASPS of other organs is also surgery if no distant metastasis has developed and resection of the primary lesion is applicable [6]. According to a previous report, treatment options for ASPS patients for whom surgery is not applicable or with distant metastasis or postoperative residual tumor include monotherapy or combination therapy of anticancer agents, molecular target agents, and radiation therapy. However, efficacy of those treatment options has not been clearly demonstrated [6–9].

The present case showed the alveolar-structured intense proliferation of tumor cells with a large acidophilic cytoplasm accompanied by abundant capillary vessels. Also, the patient's TFE3 results were positive, indicating a histopathological feature of ASPS. The differential diagnosis of ASPS includes carcinoma and melanoma, but lack of definite staining for epithelial and melanoma markers rules out these possibilities. Granular cell tumor may exhibit morphologies similar to ASPS, but these cells are invariably positive for S100. Based on those findings, we established a definite diagnosis of ASPS. Genetic testing to confirm the expression of the chimeric gene ASPL-TFE3 caused by chromosomal translocation der(17)t(X;17)(p11:q25) is a useful indicator for a definitive diagnosis of ASPS [10]. However, we were unable to obtain consent from our patient's family for genetic testing.

## 4. Conclusion

Complete resection of tumor is the only definitive treatment for ASPS. The efficacy of molecular target agents, anticancer agents, and radiation therapy has not been clearly demonstrated. Although the residual tumor was not observed in the removed bladder or urethra in the present case, we believe that cystourethrectomy was an appropriate treatment because the prognosis would have been poor if the residual tumor had been present. In pediatric patients presenting with asymptomatic macroscopic hematuria, the possibility of primary ASPS of the bladder should be considered, even though the incidence may be low.

Since the course is indolent with 18% survival at 20 years, strict follow-up is mandatory. We might consider opting a follow-up with cystoscopy or a second TUR in similar case.

## Figures and Tables

**Table 1:** Four adult cases of primary bladder ASPS previously reported.

patient	age	sex	chief complaint	treatment	local recurrence	metastases	follow-up	outcome
case 1 [2]	31	male	hematuria	partial cystectomy	+	unknown	17 months	death
			urinary frequency					
case 2 [3]	37	male	narrow urinary stream	unknown	unknown	unknown	unknown	unknown
case 3 [4]	25	female	hematuria	TUR-Bt	−	−	45 months	alive
			dysuria					
case 4 [5]	18	male	macroscopic hematuria	TUR-Bt	−	−	28 months	alive
			dysuria	partial cystectomy				

TUR-Bt, transurethral resection of the bladder tumor.
